# Neonatal emollient therapy and massage practices in Africa: a scoping review

**DOI:** 10.1093/inthealth/ihad052

**Published:** 2023-07-22

**Authors:** Keona J H Blanks, Milton W Musaba, Lily Ren, Kathy Burgoine, David Mukunya, Andrew Clarke, Sarah Williams, Tewodros Gebremichael, Peter Waiswa, Gary L Darmstadt

**Affiliations:** Stanford University, 450 Serra Mall, Stanford, CA 94305, USA; Department of Obstetrics and Gynaecology, Busitema University Faculty of Health Sciences, Pallisa, Mbale, PO Box 1460, Uganda; Lane Medical Library, Stanford Medicine, Stanford University, 300 Pasteur Drive, L109, Stanford, CA 94305, USA; Mbale Clinical Research Institute, Plot 29, 33 Pallisa, Mbale, Uganda; Busitema University Faculty of Health Sciences, Pallisa, Mbale, PO Box 1460, Uganda; Sanyu Africa Research Institute, Mbale, PO Box 2190, Uganda; Global Programs, Save the Children UK, 1 St John's Lane, London EC1M 4AR, UK; Global Programs, Save the Children UK, 1 St John's Lane, London EC1M 4AR, UK; Global Programs, Save the Children UK, 1 St John's Lane, London EC1M 4AR, UK; Makerere University School of Public Health, College of Health Sciences, Plot 1 New Mulago Hospital Complex, Kampala, Uganda; Prematurity Research Center, Department of Pediatrics, Stanford University School of Medicine, 1701 Page Mill Road, Palo Alto, CA 94304, USA

**Keywords:** Africa, bathing, emollient, massage, newborn health, skin care

## Abstract

There have been few reports from Africa on the use and health effects of emollient therapy for newborn infants. We aimed to describe neonatal skin care practices in Africa, and to illuminate opportunities to introduce evidence-based interventions to improve these practices. We conducted a scoping review of the quantitative and qualitative published peer-reviewed and grey literature in English on emollient use in Africa. Outcomes of interest included neonatal skin care practices, with a focus on the application of oils and other products to infant skin, including in association with bathing and massage. We screened 5257 articles and summarised findings from 23 studies—13 qualitative, nine quantitative and one mixed methods—that met our study criteria. Seven studies reported the use of emollients for perceived benefits, including thermal care, treatment for illness, promotion of growth and development, infection reduction, skin condition improvement, spirituality and lubrication to aid massage. Four studies reported the quantitative health impact of skin care product applications, including improvements in skin condition, neurodevelopment and bone growth, as well as a reduction in nosocomial infections. This review highlights opportunities for skin care intervention and future research on neonatal skin care practices in Africa.

## Introduction

Most neonatal deaths (98%) occur in low- and middle-income countries and are largely ascribed to complications from preterm birth (birth before 37 completed weeks of pregnancy), intrapartum-related events, serious infections and congenital anomalies.^1^ At the regional level, sub-Saharan Africa (SSA) had the highest neonatal mortality rate in the world in 2019 at 27 deaths per 1000 live births.^2^

Compromised skin barrier function is an important factor associated with preterm birth.^3^ The skin barrier of preterm infants is developmentally immature, has a minimal protective layer of vernix and heightened susceptibility to injury, and is functionally compromised, leading to increased losses of water, heat and energy and invasion of pathogens through the skin. These processes contribute to impairment of growth and neurodevelopment and an increased risk of mortality.^3,4^ Risk is further magnified in low-resource settings in contexts of maternal malnutrition and unsanitary environments.^5–8^

Emerging evidence suggests that emollient therapy promotes postnatal growth, reduces hospital-acquired infections and has the potential to reduce mortality and enhance neurodevelopmental outcomes.^8–15^ The WHO recently reviewed the evidence for emollient therapy and applied the DECIDE—Developing and Evaluating Communication strategies to support Informed Decisions and practice based on Evidence—framework, including an assessment of health effects (benefits, harms), certainty of the evidence, values of families and health workers, acceptability, resource requirements, feasibility and equity. This resulted in a conditional recommendation to consider the use of emollient therapy in preterm or low birthweight (LBW) infants globally, and a call for additional research, particularly in Africa.^16–18^ While the application of oils and other products to the skin of newborn infants is a widespread cultural practice in South Asia,^19–21^ less is known about this behaviour in Africa,^22^ as there have been few reports from Africa on the effects of emollient therapy on newborn health.^12^

The main objective of this scoping review was to describe neonatal skin care practices in Africa, and to illuminate opportunities to introduce evidence-based interventions to improve these practices. We aimed to address two primary research questions: ‘What are the common neonatal skin care practices throughout Africa, with a focus on bathing, the application of oils and other products, and massage?’ and ‘What is the reported impact of neonatal skin care practices throughout Africa, for example, the impact of emollient therapy on neonatal survival, growth, infection and neurodevelopment?’^23^

## Methods

### Review framework

The review followed a methodological framework proposed by Arksey and O'Malley,^24^ consisting of the following five steps: (i) identifying the research question(s); (ii) identifying relevant studies; (iii) selection of eligible studies; (iv) charting the data; and (v) collating and summarising the results. A scoping review methodology was selected for its aims to delineate and identify gaps in available evidence on the area of focus. Quality appraisal was not performed. The review protocol was published previously.^23^

### Search strategy

We searched for literature pertaining to the research questions in electronic databases—PubMed, Scopus (Elsevier), Embase (Elsevier), Web of Science (Clarivate Analytics) and PsycINFO (Ovid)—published between 1 January 2000 and 15 July 2021. We initially designed a search strategy for PubMed using relevant keywords and subject headings related to skin care practices for newborn infants in Africa, as described in the review protocol.^23^ The search strategy was piloted to check the appropriateness of keywords and databases. Once finalised, the strategy was adapted for replication in the other databases, and all searches were updated on 2 September 2021, as shown in the Online Supplementary Material. The bibliographic search was supplemented with examination of the grey literature in OpenGrey, GreyNet and trial registries including ClinicalTrials.gov and the Pan African Clinical Trials Registry. Grey literature was also identified through direct queries to authors of the included literature to explore whether they were aware of unpublished literature on the topic. Finally, a ‘snowballing’ method was used to identify potentially relevant literature that was cited in the included studies.

### Selection of studies

Title and abstract and full text screening was conducted by two researchers, who each reviewed one-half of the articles. Data extraction was performed by both researchers and any discrepancies were resolved by discussion together with a third researcher. The proportion of data where there was a discrepancy was <5%. Title and abstract screening was guided by a series of eligibility criteria to ensure that the content of the included studies was relevant to the research questions. The inclusion criteria were: (1) qualitative and quantitative studies published after 1 January 2000 and prior to 1 July 2021 (updated to 2 September 2021); (2) participants were newborn infants in Africa; (3) interventions were massage, use of body oil or emollient, and bathing; and (4) outcomes were skin care practices and measures of health. Studies were excluded if they had any of the following characteristics: (1) they did not include participants from Africa; (2) were multi-centre or multi-country studies reporting data from Africa that could not be isolated from mixed summary data that included non-African countries; (3) they were studies with an exclusive focus on umbilical cord care and/or bathing practices, and an absence of information on emollient applications to the skin; (4) they were not available in English or French; and (5) the full text of the article could not be obtained. Screening processes were aided by COVIDENCE software (Melbourne, Australia).

### Data extraction and management

We used a series of guiding queries to extract relevant data. Because most of the studies that were relevant to this review were qualitative or observational in design (interviews, focus group discussions and surveys), this approach was selected in lieu of the Population, Exposure, Comparator and Outcomes framework. This same approach was applied to randomised controlled trials (RCTs) and quasi experimental clinical trials.

We present findings for oil/emollient use and massage as the primary outcomes. Data were recorded on bathing, primarily in relation to emollient and massage practices. Because the review was not designed to comprehensively identify newborn bathing practices in Africa, we did not summarise these data separately.

For emollient use, data on the following outcomes were extracted and summarised: sample size; location of the study (city/district/province/country and hospital/home setting); gestational age and chronological age of infants; type of substance(s) applied to the skin; whether the substance(s) was/were applied to the umbilical cord; how often substance(s) was/were applied; by whom was/were the substance(s) applied; how was/were the substance(s) applied; how was/were the substance(s) distributed on the body (i.e. the scalp, nappy area, etc.); health impacts of product application; acceptability of the product(s) to those involved in the study; product preferences of those involved in the study; why the substance(s) was/were applied; with what were the newborns bathed; temperature of the bath water; whether anything was applied to the skin after bathing, and if so, what substance(s) were applied; by whom were the newborns bathed; how often were the newborns bathed; why were the newborns bathed in a particular way; the mode of childbirth if recorded (i.e. spontaneous vaginal delivery/caesarean section); health outcome measures, if any; observations about newborns’ responses to care; and descriptive or injunctive norms and/or perceived sanctions of norm(s), if any, that influenced the scenario. Regarding massage, the following data were extracted and summarised: type of massage performed; how massage was performed; how often massage was performed; when massage was performed; who performed the massage; the rationale behind the selected massage technique; perceived benefits of the technique; any harms or concerns about the technique; and health impacts of the massage technique.

### Patient and public involvement

Patients and/or the public were not involved in the design, conduct, reporting or dissemination plans for this research.

## Results

**Figure 1.**
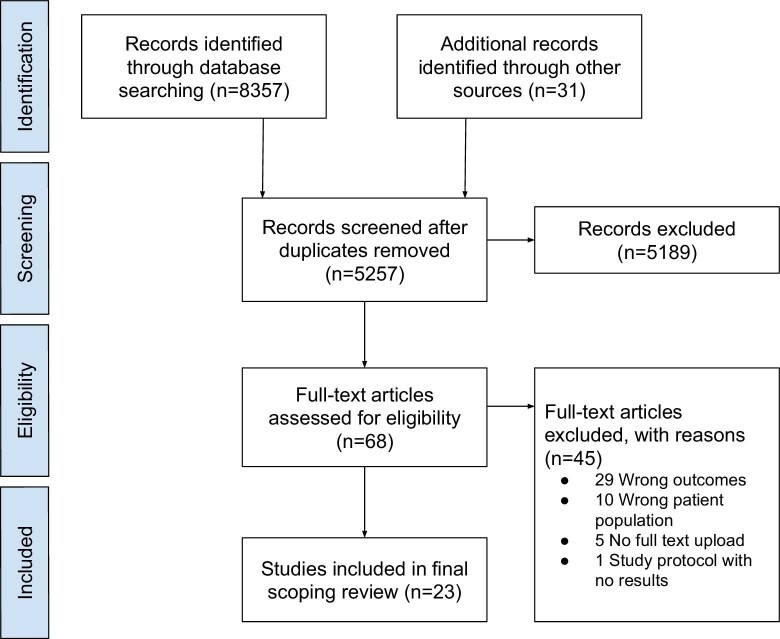
PRISMA flow diagram for the scoping review on newborn skin care practices in Africa.

We imported 8357 studies and 31 records from other sources for screening, from which 3131 duplicates were removed (Figure 1). The remaining 5257 studies underwent title and abstract screening, of which 5189 studies were deemed irrelevant. We assessed the remaining 68 full-text studies for eligibility; 45 studies were excluded and 23 studies were included.

Data were extracted from 23 qualitative and quantitative studies conducted in communities and health facilities across Africa (Table 1).^12,25–46^ Data came from nine countries: South Africa, Zambia, Tanzania, Kenya, Uganda, Ethiopia, Ghana, Nigeria and Egypt. Thirteen studies were qualitative in nature with mothers, relatives and herbal sellers as key subjects of interviews, focus group discussions and/or surveys about newborn infants. Nine studies were quantitative with neonatal subjects; and one study with newborn-maternal pairing was both qualitative and quantitative, including newborn subjects for the quantitative component and mothers for the qualitative inquiry. Of the seven studies that reported the gestational age of infant participants, five studies focused on preterm infants (<34 wk [n=1], 28–34 wk [n=1], <37 wk [n=1], 28–37 wk [n=1], 30 wk [n=1]) and two studies included preterm and term infants (mean age of 39.7 wk [n=1], 28–41 wk [n=1]). Nine studies were community-based, nine were hospital-based, four were a combination of the two and one was home- and work-based. Of the hospital-based studies, facilities were primary care clinics (n=2), district-level hospitals (n=5) and referral hospitals (n=2).

**Table 1.** Characteristics of included studies on neonatal emollient therapy and massage practices in Africa

Author and date	Title of study	Aims	Location of study	Setting of study	Sample size	Gestational age of the neonates	Chronological age of the neonates
Byaruhanga et al., 2011^25^	Hurdles and opportunities for newborn care in rural Uganda	To explore the acceptability and feasibility of newborn care practices at household and family level in the rural communities in different regions of Uganda with regards to birth asphyxia, thermo-protection and cord care	Uganda	Community	Qualitative: 9 FGDs with mothers, 6 in-depth interviews with TBAs	Not stated	Not stated
Erinoso et al., 2016^26^	Herbal recipes used for the traditional management of infantile dermatitis in Odeda, southwestern Nigeria	To investigate the plants used in the traditional management of infantile dermatitis and other neonatal skin infections with emphasis on the role of spices	Nigeria, Ogun state	Community	Qualitative: structured questionnaires (and personal interviews) were administered to 36 nursing mothers, 30 herbal sellers	Not stated	No neonates involved
Mrisho et al., 2008^27^	Understanding home-based neonatal care practices in rural southern Tanzania	To provide a basis for the development of strategies for improving neonatal survival	Tanzania	Community	Qualitative: 40 in-depth interviews, 16 FGDs	Not stated	Not stated
Darmstadt et al., 2004^12^	Topically applied sunflower seed oil prevents invasive bacterial infections in preterm infants in Egypt	To test the impact of topical application of sunflower seed oil 3 times daily	Cairo, Egypt	NICU	103	<34 wk	<72 h old
Adejuyigbe et al., 2015^28^	‘Why not bathe the baby today?’: A qualitative study of thermal care beliefs and practices in four African sites	To examine beliefs and practices related to neonatal thermal care in three African countries	Multi-site, Northern Nigeria, Lindi and Mwata, Tanzania, and Oromia, Ethiopia	Home and workplace	Qualitative: 16–20 narrative interviews with mothers, 8 observations of neonatal bathing, in-depth interviews with 12–16 mothers, 9–12 grandmothers, 8 health workers and 0–12 birth attendants (numbers varied by site)	Not stated	Not stated
Perez et al., 2015^29^	Massage therapy improves the development of HIV-exposed infants living in a low socio-economic, peri-urban community of South Africa	To assess the effect of massage therapy on the growth and development of infants of HIV-infected mothers in a low socioeconomic community in Cape Town	Cape Town, South Africa	Primary healthcare clinic	161	Not stated	<6 wk
Moussa et al., 2021^30^	Effect of infant massage on salivary oxytocin level of mothers and infants with normal and disordered bonding	To assess the effect of infant massage on salivary oxytocin level of mothers and their infants during the postpartum period	Cairo, Egypt	Primary healthcare clinic	37 pairs of infants and mothers	Not stated	2 mo
Aly et al., 2004^31^	Physical activity combined with massage improves bone mineralization in premature infants: A randomized trial	To test the hypothesis that massage combined with physical activity can stimulate bone formation and ameliorate bone resorption in premature infants	Cairo, Egypt	NICU	30	28–34 wk	Not stated
Sacks et al., 2015^32^	Skin, thermal and umbilical cord care practices for neonates in southern, rural Zambia: a qualitative study	To understand local practices during the postnatal period	Zambia, Chomba District	Community and hospital	Qualitative: 36 in-depth interviews, 5 FGDs, 8 observational sessions with recently delivered women, TBAs, and clinic and hospital staff from three sites	Not stated	Not stated
Kayom et al., 2015^33^	Newborn care practices among mother-infant dyads in urban Uganda	To understand newborn care practices in this urban community	Kampala, Uganda	Community	Quantitative: 325 neonates Qualitative: 9 FGDs	Mean gestational age 39.7 (SD 2.3) wk	Not stated
Buser et al., 2021^34^	Maternal knowledge of essential newborn care in rural Zambia	To compare maternal knowledge of newborn care for women referred from facilities with and without maternity waiting homes	Zambia	Hospital	Qualitative: a two-group comparison design was employed using a face-to-face survey with a convenience sample, 250 mothers	Not stated	15–24 y of age
Ogunlesi et al., 2009^35^	Prevalence and risk factors for hypothermia on admission in Nigerian babies <72 h of age	To determine the prevalence and risk factors for neonatal hypothermia at admission in the first 72 h of life	Nigeria	Tertiary hospital with neonatal services	111	36.9±3.2 (range: 28–41) wk	Newborn infants between 0 and 68 h of age (mean±SD of 13.6±18.9 h)
Tette et al., 2020^36^	The profile, health seeking behavior, referral patterns, and outcome of outborn neonates admitted to a district and regional hospital in the Upper West Region of Ghana: A cross-sectional study	To examine the health practices, care-seeking behaviour and referral of sick outborn neonates to a district and regional hospital in the Upper West region of Ghana	Ghana	Hospital	153	<37 wk (n=26), ≥37 wk (n=127)	Not stated
Thairu and Pelto, 2008^37^	Newborn care practices in Pemba Island (Tanzania) and their implications for newborn health and survival	To describe newborn care practices and their potential impact on newborn health	Pemba Island of Zanzibar, Tanzania	Community	12 mothers provided descriptive data; 26 mothers reported actual practice	Not stated	Not stated
Okyere et al., 2010^38^	Newborn care: the effect of a traditional illness, asram, in Ghana	To explore the role of a traditional illness of the newborn, asram, in care-seeking in rural Ghana	Ghana	Community	25 birth narratives, 30 in-depth interviews, 2 FGDs with recently delivered/pregnant women; 20 in-depth interviews, 6 FGDs with birth attendants/ grandmothers; 12 in-depth interviews, 2 FGDs with husbands; 6 in-depth interviews with asram healers	Not stated	Delivered in the last 2 mo
Amare et al., 2015^39^	Current neonatal skin care practices in four African sites	To assess skin care practices and emollient use in four African sites	Ethiopia, Tanzania, Borno, Ekiti	Community	Newborn care narratives with 16–20 mothers who delivered in the past 3 mo; observations of 8 babies <1 mo of age; in-depth interviews with families of children <1 y of age (12–16 mothers, 9–12 grandmothers, 8 fathers); in-depth interviews with 8 health workers and 0–12 birth attendants; in-depth interviews with 8–10 emollient sellers; FGDs with mothers and grandmothers of children <1 y of age, health workers and birth attendants: 4 with mothers, 4 with grandmothers, 0–2 with health workers and 0–4 with birth attendants	Not stated	Not stated
Waiswa et al., 2010^40^	‘I never thought that this baby would survive; I thought that it would die any time’: perceptions and care for preterm babies in eastern Uganda	To explore the current care for and perceptions about preterm babies among community members in eastern Uganda	(Iganga and Mayuge) in eastern Uganda	Community and health facilities	In-depth interviews: 11 CHWs, 10 mothers, 6 fathers, 3 grandmothers. Three FGDs with midwives, women and men	Preterm infants	Not stated
Darmstadt et al., 2007^41^	Neonatal home care practices in rural Egypt during the first week of life	To provide information about home care practices for newborns in rural Egypt, in order to improve neonatal home care through preventive measures and prompt recognition of danger signs	Aswan (Upper Egypt), Luxor (Upper Egypt) and Fayoum (Lower Egypt)	Community	217 households	Not stated	Not stated
Mullany et al., 2009^42^	Incidence and risk factors for newborn umbilical cord infections on Pemba Island, Zanzibar, Tanzania	To assess incidence and risk factors for newborn umbilical cord infections on Pemba Island, Zanzibar, Tanzania	Pemba Island of Zanzibar, Tanzania	Community and health facilities	9550 cord assessments in 1653 infants	Not stated	Not stated
Adejuyigbe et al., 2008^43^	Feeding and care of low-birthweight babies in two rural communities in south-western Nigeria	To highlight the sociocultural beliefs and practices relating to the care and feeding of LBW babies in two rural communities in the south-west of Nigeria	Southwestern Nigeria	Community	30 LBW, 30 normal birthweight infants	Not stated	Not stated
Nyaga et al., 2021^44^	Effect of massage therapy on preterm neonate's body temperature	To determine the effect of massage therapy on the body temperature of preterm neonates	Western Kenya	Hospital NICU level II	72 infants	28–37 wk	Not stated
Waiswa et al., 2008^45^	Acceptability of evidence-based neonatal care practices in rural Uganda—implications for programming	To explore the acceptability of these interventions in two rural districts of Uganda	(Iganga and Mayuge) in eastern Uganda	Community and health facilities	In-depth interviews: 11 CHWs, 10 mothers, 6 fathers, 3 grandmothers; 3 FGDs with midwives, women and men	Preterm infants	Not stated
Dramowski et al., 2021^46^	Impact of 1% chlorhexidine gluconate bathing and emollient application on bacterial pathogen colonization dynamics in hospitalized preterm neonates: A pilot clinical trial	To assess the impact of 1% chlorhexidine gluconate bathing and emollient application on bacterial pathogen colonisation dynamics in hospitalised preterm neonates	Cape Town, South Africa	Tertiary hospital	80 preterm infants equally divided into 4 arms	30 wk	1–4 d postnatal

Abbreviations: CHW, community health worker; FGD, focus group discussion; LBW, low birthweight; NICU, neonatal ICU; TBA, traditional birth attendant.

Unadulterated emollients were utilised in seven studies, with sunflower seed oil (SSO; n=2) and coconut oil (n=3) as key emollients (Table 2). Emollients were adulterated through mixture with herbal treatment or heating in eight studies. For newborns in rural Zambia who were perceived to be small, leaves from the mabono plant were mashed and mixed into petroleum jelly and placed directly on the skin, all over the newborn infant's body.^32^

**Table 2.** Newborn skin care practices in Africa

	Bathing	Cord care	Oiling/massage	Who administered oiling/massage	Massage technique, if performed	How often oiling/massage was administered	When oiling/massage was administered	Health impacts of oiling/massage product application
Uganda	Bathed with herbal water (water mixed with local herbal medication composed of a mixture of several leaves boiled together called ‘ekyogero or eshabiko’),^25^ used warm wet wipes^40^ Bathed with ‘ekyogero’, a mixture of various herbs including leaves, roots and tree barks^33^	Salty water, powder, vaseline, spirit, normal saline, ripe banana (gonja), sap, soot, ash, saliva and herbs applied exclusively to the cord^33^	Unspecified local herbs for bathing and applying to infants’ skin^25^ Smeared baby with cooking oil using a clean cloth^40^	Not stated^25^ Mother^33,40^	Not stated^25,33,40^	Not stated^25^ Twice a day^33^	Not stated^25,33,45^ Always^40^	Not stated^25,33,45^ Makes skin of the preterm infant strong^40^
Tanzania	Traditional herbs mixed with either cooking oil or water, SSO, coconut oil, or other oil^27^ Not stated^28^ Plastic disc, water^42^	Sesame or other cooking oil, seed oil from a local tree Traditional herbs mixed with either cooking oil or water that has been used to wash an adult woman's genitals (numbati) is applied to heal the cord^27^ Not stated^28^ Charcoal^37^	Traditional herbs mixed with either cooking oil or water, SSO, coconut oil or other oil^27^ Unspecified oil (‘She puts heavy clothes, socks, hat and she warms oil…and rubs on the body of the baby…also she bathes the baby with warm water’)^28^ Olive oil, coconut oil, oil mixed with traditional medicine^37^ Coconut oil^43^ Coconut oil, cooking oil, baby oil^39^ Coconut oil^42^	Traditional birth attendants, relatives, other birth assistants^27^ Not stated^28^ Mothers^37,39^	Not stated^27^ Not stated^28^ After applying oil onto their palms, mothers warm them over an open fire, and quickly massage the newborn all over^37^	Not stated^27^ Not stated^28^ Twice a day: morning and evening^37^ After every birth^39^	Not stated^27^ Not stated^28^ Practices in the early postpartum period: (1) wash baby with water; (2) wash baby with soap; (3) apply oil on baby's skin; (4) apply olive oil on baby's skin; (5) apply oil on baby's fontanel; (6) massage baby after warming hands; (7) massage baby with coconut oil; (8) carry baby to ‘pass the doorway’ 7 d after birth^37^ After every birth^39^	Protects neonate from evil spirits^27^ Not stated^28^ Practices for preventing and managing illness Massaging babies with coconut oil helps their limbs and joints ‘to develop’^37^ EMs were applied to make the skin ‘soft’, ‘smooth’, ‘attractive’, ‘healthy’, ‘strong’ and ‘rash free’, keeps the baby warm, softens/strengthens the joints/bones, shapens the baby, ensures flexibility, encourages growth and weight gain, and helps the baby sleep^39^ Cord infection^42^
South Africa	Not stated^29^ 1% aqueous CHG, 1% CHG+EM, EM only and SOC (no antiseptic/EM)^46^	Not stated^29^ 1% aqueous CHG, 1% CHG+EM, EM only and SOC (no antiseptic/EM)^46^	Plain carrier massage oil (usually jojoba oil) with no essential oils added^29^ 1% aqueous CHG, 1% CHG+EM, EM only and SOC (no antiseptic/EM) [whole body])^46^	Not explicitly stated but probably, health worker^46^	Yes^29^ Not stated^46^	Once daily for 15 min^29^ Once daily for 10 d post enrolment.^46^	Not stated^29^ 1–4 d postnatal^46^	Daily 1% CHG bathing significantly reduced overall bacterial colonisation density No CHG-related adverse events occurred EM application significantly improved skin condition but was associated with higher rates of *Staphylococcus aureus* colonisation via neonatal skin colonization)^46^ Improved the overall development and had a significant effect on the hearing and speech and general quotient of HIV-exposed infants (via mean difference in scores, based on the Griffiths Scales (growth, development, hearing, speech)^29^
Nigeria, Ogun state ^26^ Nigeria ^35^ South Western Nigeria^43^ Borno and Ekiti ^39^ Northern Nigeria ^28^	Bathing with warm extracts of the plants and the use of coconut oil as cream, Shea butter, black soap,^26^ water,^28^ water and herbal mixtures^43^	Not stated^26^ Mentholatum,^39^ herbal dressing^43^	Coconut oil, Aframomum melegueta leaf, Cajanus cajan leaf, and Zingiber officinale leaf (the plants are ground, and worked into Shea butter, and used as cream to massage the affected area)^26^ Unspecified oil^35^ Ground nut oil for removing vernix^28^ Borno: ground nut oil to clean the baby after delivery Other oil to clean the baby after delivery, Shea butter, baby oil, ‘olive oil, mahogony or neem oil to the fontanelle Engine oil on circumcision wound Ekiti: Goya ‘olive’ oil to clean baby after delivery, other oil to clean baby after delivery, baby lotions, herbal/medicated creams, Shea butter, mentholatum, engine oil, baby oil to the fontanelle^39^ Not stated^43^	Mother^26,39^ Not stated^43^	Apply to affected area^26^ Not stated^43^	Not stated^26^ After every birth^39^ Not stated^43^	After bathing^26^ After bathing^35,39^ Not stated^43^	Treat various skin ailments, traditional management practice^26^ Not stated^28,35^ EMs were applied to make the skin ‘soft’, ‘smooth’, ‘attractive’, ‘healthy’, ‘strong’ and ‘rash free’, to keep the baby warm, to soften/strengthen the joints/bones, shape the baby, ensure flexibility, encourage growth and weight gain, and to help the baby sleep^39^ Not stated^43^
Zambia	Water^32,34^	Umbilical cord: powders made of roots, burnt gourds or ash^32^ Petroleum jelly, glycerin, cooking oil^34^	Whole body but taking care not to involve the mouth, ears and eyes Petroleum jelly, commercial baby lotion, cooking oil (mixture, similar to vegetable oil, often made with SSO), leaves from the mabono plant mashed into petroleum jelly^32^ Not stated^34^	Not stated^32^ Not stated^34^	Whole body but taking care not to involve the mouth, ears and eyes^32^ Not stated^34^	Several times^32^ Not stated^34^	Mothers and TBAs^32^ Not stated^34^	To ward off malevolent spirits, improve babies skin, keep baby warm with mabono leaves^32^ Not stated^34^
Kenya	Not stated^44^	Not stated^44^	Massage^44^	Nurse^44^	Not stated^44^	Three times a day^44^	Not stated^44^	Effect on body temperature^44^
Egypt	Not stated^12,30,31^ Soap and water^41^	Not stated^12,30,31^ Alcohol, kohl (local)^41^	SSO^12,41^ Massage^30,31^	Not stated^12,41^ Mother^30^	Whole body^12,31^ Tappans technique for massage^30^ Not stated^31^ Not done^41^	Three times daily for the first 14 d and then twice daily until 28 d of life or until discharge from the NICU^12^ Once daily^31^ 1–3 times daily^41^	Maximised time between oil application and bathing^12^ At each clinic visit^30^ Not stated^31,41^	Significant improvement in skin condition and a highly significant reduction in the incidence of nosocomial infections (i.e. noncontaminated positive blood or cerebrospinal fluid culture for suspected sepsis after the first 48 h of hospitalisation)^12^ Oxytocin levels in saliva, infant massage increased salivary oxytocin level in mothers and infants with normal bonding and it had no effect on salivary oxytocin level of baby^30^ Improved bone growth and physical activity^31^ Not stated^41^
Ghana	Herbal water^36^ Herbal baths^38^	Breast milk, applied toothpaste to cord, used powder, used cow dung^36^ Not stated^38^	Shea butter, palm kernel oil, herbal enema, herbal lotions/creams, salt water^36^ Not done^38^	Not stated^36^	Not done^36,38^	After bathing^36^ Bathed at least two or three times a day for at least the first week More often if the infant is severely ill^38^	After bathing^36^ Not stated^38^	‘Cultural traditions and societal factors’^36^ To obtain cure for illness^38^
Ethiopia	Not stated^39^	Not stated^39^	Butter, vaseline, hair lotion^39^	Mothers/care takers^39^	Done during the application Technique sometimes rough and included rubbing, pulling, pressing, manipulating joints and shaping features^39^	Once daily^39^	Only after the morning bath^39^	To make the skin ‘soft’, ‘smooth’, ‘attractive’, ‘healthy’, ‘strong’ and ‘rash free’,to keep the baby warm, to soften/strengthen the joints/bones, shape the baby, ensure flexibility, encourage growth and weight gain, and to help the baby sleep^39^

Abbreviations: CHG, chlorhexidine gluconate; EM, emollient; NICU, neonatal ICU; SOC, standard of care; SSO, sunflower seed oil; TBA, traditional birth attendant.

Various oils were reported in eight of nine countries, in addition to traditional herbs in Uganda and Tanzania. Use of butter, vaseline and hair lotion was reported in Ethiopia. The skin care practices were administered by mothers, relatives, traditional birth assistants or nurses in all countries. Application involved massage in sites in Ethiopia, Egypt, Zambia, South Africa and Tanzania. Across sites, the frequency of emollient application varied; emollient was applied at frequencies ranging from once exclusively after birth to three times daily.

Four studies reported quantitative findings on the health impact of skin care product applications. These findings included: a significant improvement in skin condition (p=0.037) and a highly significant reduction in the incidence of nosocomial infections (adjusted incidence rate ratio, 0.46; 95% CI 0.26 to 0.81; p=0.007);^12^ an improvement in overall neurodevelopment and a significant effect on the hearing and speech and general quotient percentile (19.3 vs. 7.7) (p=0.03) based on the Griffiths Mental Development Scales;^29^ an improvement in bone growth and physical activity;^31^ and a significant improvement in skin condition and association with higher rates of *Staphylococcus aureus* colonisation.^46^

Qualitative studies in rural Nigeria, rural Tanzania, urban Tanzania, rural Zambia and rural Ethiopia examined perceptions, beliefs and norms motivating skin care practices (Table 3). Seven studies reported the use of emollients for perceived benefits, including thermal care, treatment for illness, promotion of growth and development, infection reduction, skin condition improvement, spirituality and lubrication to aid massage. Within the seven studies, thermal care was reported as the rationale for skin care practices in five sites, growth and development and improving skin condition were recorded in four sites and treating illnesses and spiritual beliefs were recorded in three sites.

**Table 3.** Beliefs and norms motivating newborn skin care practices in Africa

	Thermal care	Treatment for illness	Growth and development	Reduce infection	Improve skin condition	Spiritual	Other
Urban Uganda, community-based		Bathing: the herbal medications were used to prevent and treat skin rashes (locally called ‘nnoga’) and treat abdominal colic^33^				Kyogero, to bring good fortune. Focus group discussion member said, ‘Bathing the baby in ekyogero makes the baby get good luck. Anyway other children are not bathed in it but they get their blessings from God.’^33^	
Urban and rural Nigeria, community-based	‘I keep my baby warm by rubbing oil on the whole of his body after bathing … ’ (35-y-old Bura mother, Borno)^40^	Coconut oil is used as cream to massage the affected area for management of infantile dermatitis^26^	To soften/strengthen the joints/bones, shape the baby, ensure flexibility, encourage growth and weight gain, and to help the baby sleep^40^		EMs were applied to make the skin ‘soft’, ‘smooth’, ‘attractive’, ‘healthy’, ‘strong’ and ‘rash free’. In Ekiti and Borno, oil was also used to help remove the vernix after delivery. In all sites, emollients were applied to make the skin ‘soft’, ‘smooth’, ‘attractive’, ‘healthy’, ‘strong’ and ‘rash free’^40^		
Rural Tanzania, community-based	To keep the neonate warm^27^	Preventing and managing illness^38^	‘A baby is massaged so that he develops; the baby's [joints] have not yet developed, each time the baby is massaged, his limbs become stronger.’^38^		To soften the skin^27^	Applying oil mixed with traditional medicine to the newborn's skin was perceived as a form of prevention against ‘evil’: ‘We say that the young baby is appealing to evil spirits (mash-etani) and to the supernatural (majini) […] when oil mixed with traditional medicine is applied on the baby's skin, those [evil spirits] encounter the bad smell of the oil, then it is not easy for them to harm the baby.’^38^	
Urban Tanzania, community-based (Lindi and Mtwara)	To keep the baby warm^40^		To soften/strengthen the joints/bones, shape the baby, ensure flexibility, encourage growth and weight gain. Some effects were jointly attributed to the EM and to massage during application: ‘It [stretching with oil] helps the body parts … to be sitting well and not to became weak … the oil enters inside the body and helps the bones to be soft and the baby becomes in good condition’ (35-y-old Tanzanian mother)^40^		EMs were applied to make the skin ‘soft’, ‘smooth’, ‘attractive’, ‘healthy’, ‘strong’ and ‘rash free’^40^		
Rural Zambia, community and hospital	‘The oil makes the baby warm, so I put the oil many times per day. I rub all over the body so all the skin is covered and his skin is shiny, but I always cover him again with mabono so he will stay warm and get bigger.’ (Interview, recently delivered woman)^32^					To ward off malevolent spirits^32^	
Urban South Africa, hospital							To aid massage movement^29^
Rural Ethiopia, community	To keep the baby warm^40^		To soften/strengthen the joints/bones, shape the baby, ensure flexibility, encourage growth and weight gain, and to help the baby sleep. Some effects were jointly attributed to the emollient and to massage during application: ‘We have seen that massaging with butter has allowed my baby to gain weight since she sleeps well’ (30-y-old mother Ethiopian mother)^40^		EMs were applied to make the skin ‘soft’, ‘smooth’, ‘attractive’, ‘healthy’, ‘strong’ and ‘rash free’^40^		

Abbreviation: EM, emollient.

## Discussion

We conducted a scoping review of literature reporting common neonatal skin care practices in Africa, with a focus on the application of oils and other products, and associated bathing and massage. While our search identified >5000 potential sources, only 23 were found to provide information on newborn skin care practices in Africa, pointing to the need for further research on skin care in this region.

We found oil to be a common form of emollient therapy, used in eight of nine countries included in the study. Traditional herbal mixtures in oils such as cooking oil, SSO and coconut oil were also reported as a form of emollient in Uganda and Tanzania. The use of butter, vaseline and hair lotion was reported only in Ethiopia, yet the experience of the authors suggests that the use of vaseline is common in other locations, including Kenya, Uganda and Zimbabwe. Quantitative studies that reported health outcomes found a variety of effects, including an improvement in skin condition associated with a reduction in nosocomial infections^11^ or an increase in the rates of *S. aureus* colonisation;^46^ an improvement in measures of the neurodevelopment of HIV-exposed infants;^29^ and increased growth and physical activity.^31^ A variety of beliefs and norms were reported to motivate neonatal skin care practices, including improved thermal care, treatment for illness, promotion of growth and development, infection reduction, improved skin condition, spiritual beliefs and massage to aid movement and improve strength.

Our present review adds 14 additional publications to the literature pool compared with those analysed in a previous review of the literature on neonatal skin care in Africa by Duffy et al.^22^ We found similar practices and motivations for skin care as reported by Duffy et al.^22^ For example, massage was associated with perceived benefits of increasing limb strength and suppleness in Nigeria and Tanzania and to protect and strengthen the skin, cleanse impurities and warm the baby in Uganda, Tanzania, Nigeria and Egypt. Our present review also indicates that spiritual beliefs and the belief that massage will aid movement are motivators of skin care practices in Africa. Shared themes from the two reviews for the motives behind topical emollient therapy include thermal care, treatment for illness, growth and development, infection reduction and skin condition improvement. Our review found that oil massage does not appear to be a universal practice in Africa, as it is in much of South Asia. There is a need for more formative research to understand avenues for sensitisation and introduction of emollient therapy in Africa.

Many emollients that are reportedly applied to newborn skin in South Asia and SSA have been demonstrated in laboratory studies with a mouse model of human infant skin to perturb important epidermal functions, including permeability barrier homeostasis, and thus are potentially harmful,^47,48^ highlighting the need for improved emollient practices. Given the relevance of the mouse model for human infant skin, it can be anticipated that these products perturb the epidermal permeability barrier of infants to whom they are applied, which may contribute to the high rates of neonatal morbidity and mortality seen in SSA.^49^ Intervention with appropriate products that have a compositional profile consistent with the promotion of skin barrier function (e.g. high levels of linoleic acid) plus demonstrated enhancement of skin barrier function in the mouse model is crucial for the improvement of neonatal health outcomes, especially in preterm infants whose skin barrier is highly permeable and fragile.

The use of oil as emollient therapy is highly prevalent in Asia, providing some observations that may be helpful for Africa. A study conducted in Maharashtra and Madhya Pradesh states in India found that massage was mostly conducted using oils.^50^ Like our findings from Africa, reported perceived benefits of infant massage included increased bone strength and better growth, while no harm was perceived. Improved sleep was an additional perceived benefit. In Bangladesh, a study found that topical therapy with SSO or Aquaphor was perceived by many families to be superior to traditional use of mustard oil after their infants received emollient therapy with SSO or Aquaphor in the hospital.^51^

A study conducted in Uttar Pradesh pointed to the necessity of further research on potential approaches to improving adherence to recommended therapy, particularly in community settings.^52,53^ Acceptance of SSO in the intervention arm was high at 89.3%, but adherence to exclusive applications of SSO was 30.4%.^53^ The community's inherent belief in the goodness of traditional use of mustard oil appeared to be strong, suggesting that more intensive behaviour change management was required to shift deeply entrenched community norms toward adoption of recommended practices.

The application of mustard oil is particularly universal in Asia due to its perceived benefits including prevention of infections and hypothermia, promotion of strength, maintenance of health and provision of warmth.^19–21^ There is evidence from mouse models, however, that mustard oil may be harmful to the skin.^47^ A community-based trial in Nepal comparing the effects of SSO and mustard oil on skin integrity in premature and full-term newborns suggests that SSO may be protective for newborns in lower resource settings due to the more rapid acid mantle development observed for SSO.^54^ The potential health impact for intervention with SSO applications at population level was shown in the Uttar Pradesh study where growth increased by 0.94 g/kg/d, rates of hospitalisation and of any illness were reduced by 36% and 44%, respectively, and the mortality of the subgroup of very low birthweight (≤1500 g) infants was reduced by 52%.^52,53^

A strength of the present review is its synthesis of data from quantitative and qualitative research on emollient use in SSA, thus expanding on the ‘how’ and ‘why’ of previous reports.^22^ A limitation, however, is that we did not conduct a formal quality appraisal or assessment of the risk of bias for the studies that were included, given the relatively small number of studies on our topic of interest.

Our study also highlights key areas for additional research (Table 4). While the WHO recently recommended that emollient therapy—particularly with SSO or coconut oil—should be considered for the care of preterm or LBW infants globally, it was also noted that there is a need for additional evidence in a number of areas, including impacts on growth, thermoregulation, the microbiome, invasive infection/sepsis, mortality and longer-term neurodevelopment. In addition, more data are needed on emollient composition and dosing for maximal effectiveness. Data are particularly lacking from Africa. Similar to research undertaken in South Asia, we recommend prioritisation of systematic, prospective RCTs in the most vulnerable group with the greatest skin barrier compromise—hospitalised very low birthweight infants—in several countries, using common study designs that would enable data to be included in existing meta-analyses, ideally enrolling infants with untreated skin for comparison with infants treated identically in all ways, except for application of emollient. We also note several other research questions that emerged from our study of skin care and emollient-use practices in Africa (Table 4).

**Table 4.** Key research questions on emollient therapy in newborn infants in Africa

What motivates the use and choice of skin-care products for newborn infants in sub-Saharan African contexts?
What are the survival, growth, morbidity, microbiome, thermoregulatory and neurodevelopmental impacts of emollient therapy on newborn infants in SSA?
What forms of emollient therapy are effective for newborns in sub-Saharan African contexts?
Do skin-care practices for preterm infants differ from practices for full-term infants in SSA?
What traditional neonatal skin care practices in SSA could we learn from? Which could be potentially harmful?
What values do sub-Saharan African families and healthcare providers place on skin and skin care?
How do social norms and cultural practices influence skin care practices in SSA?
Who do we need to educate for emollient interventions to be successful in SSA?
How can adherence to recommended skin care practices be increased, and use of harmful practices be diminished in SSA?

Abbreviation: SSA, sub-Saharan Africa.

## Conclusions

The present review highlights opportunities for skin care intervention and future research on neonatal skin care practices in Africa. While emollient therapy is a promising intervention to prevent serious infections and improve growth, neurodevelopmental and survival outcomes among preterm and/or LBW infants, future qualitative research, emollient intervention trials for preterm and LBW newborns in Africa, as well as implementation research, are necessary to establish the effectiveness and feasibility of widescale use and impact of emollient therapy in this region.

## Data Availability

The data underlying this article are available in the article.
